# Influence of taste disorders on dietary behaviors in cancer patients under chemotherapy

**DOI:** 10.1186/1475-2891-9-15

**Published:** 2010-03-24

**Authors:** Karla Sánchez-Lara, Ricardo Sosa-Sánchez, Dan Green-Renner, Cindy Rodríguez, Alessandro Laviano, Daniel Motola-Kuba, Oscar Arrieta

**Affiliations:** 1 Oncology Center Diana Laura Riojas de Colosio, Médica Sur Clinic and Foundation, Mexico City, Mexico; 2 Department of Medical Oncology, National Cancer Institute, Mexico City, Mexico; 3 Department of Clinical Medicine, University La Sapienza, Roma, Italia

## Abstract

**Objectives:**

To determine the relationship between energy and nutrient consumption with chemosensory changes in cancer patients under chemotherapy.

**Methods:**

We carried out a cross-sectional study, enrolling 60 subjects. Cases were defined as patients with cancer diagnosis after their second chemotherapy cycle (n = 30), and controls were subjects without cancer (n = 30). Subjective changes of taste during treatment were assessed. Food consumption habits were obtained with a food frequency questionnaire validated for Mexican population. Five different concentrations of three basic flavors --sweet (sucrose), bitter (urea), and a novel basic taste, umami (sodium glutamate)-- were used to measure detection thresholds and recognition thresholds (RT). We determine differences between energy and nutrient consumption in cases and controls and their association with taste DT and RT.

**Results:**

No demographic differences were found between groups. Cases showed higher sweet DT (6.4 vs. 4.4 μmol/ml; p = 0.03) and a higher bitter RT (100 vs. 95 μmol/ml; *p *= 0.04) than controls. Cases with sweet DT above the median showed significant lower daily energy (2,043 vs.1,586 kcal; p = 0.02), proteins (81.4 vs. 54 g/day; *p *= 0.01), carbohydrates (246 vs.192 g/day; *p *= 0.05), and zinc consumption (19 vs.11 mg/day; *p *= 0.01) compared to cases without sweet DT alteration. Cases with sweet DT and RT above median were associated with lower completion of energy requirements and consequent weight loss. There was no association between flavors DT or RT and nutrient ingestion in the control group.

**Conclusion:**

Changes of sweet DT and bitter RT in cancer patients under chemotherapy treatment were associated with lower energy and nutrient ingestion. Taste detection and recognition thresholds disorders could be important factors in malnutrition development on patients with cancer under chemotherapy treatment.

## Introduction

Malnutrition and weight loss are common in patients with cancer [1], both factors could potentially affect the response and tolerance to treatment [2,3], decrease quality of life (QOL), and associate with poor survival [4]. Multiple pathogenic mechanisms have been linked to weight loss and malnutrition in cancer patients. Modifications in smell or taste senses could play a significant role in the etiology of anorexia in cancer patients [5,6]. Taste disorders may affect food selection and contribute to poor meal intake and low quality of life [7-9]. DeWys [10] was among the first to implicate taste threshold abnormalities in anorexia development in patients with cancer. Specific mechanisms remain uncertain but some studies have associated anorexia in cancer patients with mucositis, dental disease, tumor invasion, vitamin deficiencies, poor oral hygiene, bacterial, viral or fungal oral/nasal cavity infections, postnasal drip, gastroesophageal reflux and volatile compounds in inhaled air [11-13]. Taste disorders are common in patients with cancer under chemotherapy [14-17]. Chemotherapeutic drugs associated with taste changes include cisplatin, carboplatin, cyclophosphamide, doxorubicin, 5-flourouracil, and methotrexate [18,19]. Chemotherapy drugs could affect other rapidly growing cells such as the taste receptors. A variety of circulating drugs that permeate into saliva and diffused from blood to taste receptors resulting in a phenomenon called venous taste change [20].

Previous studies have reported alterations of the four basic flavors (sweet, salty, sour and bitter) in cancer patients [21,22]; the most common complain during cytotoxic drugs administration occurs in bitter and sweet flavor recognition [16]. However, there is not enough data to determine changes in umami, a recently described basic flavor, in chemotherapy treated cancer patients [23-27].

This study evaluated self-perceived taste function exam in patients with cancer diagnosis under chemotherapy (cases) and healthy subjects (controls) to determine differences in energy intake and taste recognition.

## Patients and Methods

We conducted a cross-sectional study in a University hospital, oncology center of Mexico City. Our population consisted of 60 subjects. Exclusion criteria included history of oral/nasal cavity infections, brain disease, acute respiratory illness, gastroesophageal reflux, central nervous system metastasis, gastrointestinal cancer, and head and neck cancer. Thirty subjects with a histological diagnosis of a malignant neoplasia (breast, lung, prostate, multiple myeloma and lymphoma) after their second chemotherapy cycle were defined as cases. Thirty patient relatives were defined as controls; they were healthy and have no clinical history of acute and chronic disease. Eligible patients had histological proved diagnosis of clinical stages II and III cancer with an Eastern Cooperative Group performance status (ECOG) of 0 to 1. The study protocol was approved by the Institutional Ethics Committee. All subjects provided written informed consent.

### Physical exam

Body weight was measured in light clothing and without shoes, to the nearest 0.10 kg. Height was measured to the nearest 0.5 cm. Body mass index (BMI) was calculated in all subjects.

### Questionnaire

Subjects completed all information on demographic data: age, gender, alcohol consumption, and smoking habits. Other medications used in cases prior to this study were identified to discard those that might affect taste detection or appetite.

### Dietary history questionnaire

Patients were requested to complete a questionnaire of symptoms related to appetite loss and changes in flavor detection. Nutrients intake was evaluated using the "SNUT" program. This food frequency questionnaire (FFQ) was developed and validated for Mexican population by the National Institute of Public Health [28] SNUT is composed of a matrix listing 116 food items and 10 frequencies of consumption with specified size portion; the program also included information concerning the frequency of ingestion and the brand of vitamin supplements. SNUT software calculate daily intake of: calories, proteins, carbohydrates, saturated, polyunsaturated and monounsaturated fat; vitamins, and zinc. We calculated individual total metabolic rate (30 kcal per kilogram body weight per day) and protein requirements (1.0 gram per kilogram per day) in all patients, and compared this result with calories and protein consumption. Patients with cancer diagnosis (cases) were classified as subjects that completed their calculated needs and subjects who did not. Each of this group classification was compared looking for differences in taste perception detection threshold and recognition threshold (RT).

### Taste evaluations

Five concentrations of the three main flavor substances were dissolved in distilled water, including sucrose (3.5-15.5 μmol/ml), urea (91-115 μmol/ml), and sodium glutamate (0.3-2.7 μmol/ml). Solutions were freshly prepared every week by serial dilutions and stored at 4-5°C. Before testing, 5-ml samples in 30-ml plastic cups were brought to room temperature (24 ± 2°C); the presentation order of the five concentrations was randomized before giving them to study subjects. The patients were scheduled for study testing 4 hours after eating or smoking. The patient's mouth was rinsed with a sip of distilled water prior to testing each 5-ml sample.

### Data analysis

For descriptive purposes continuous variables were summarized as median and standard deviation and categorical variables comprised proportions. Interferential comparisons were carried out by Student t and Mann-Whitney U test according to distribution (normal and non-normal) determined by the Kolmogorov-Smirnov test. Chi square test and Fisher's Exact test were utilized to assess significance among categorical variables. When the control and test group were matched, we use the Wilcoxon Signed Ranks test and express the result like z (Mean Rank differences). Statical significance was determined as p < 0.05 with a two-sided test. All statistical analyses were carried out with SPSS/PC v. 15.0 program software (SPSS, Inc., Chicago, IL, USA).

## Results

Sixty subjects were included in the study: 30 cancer patients after their second chemotherapy cycle and 30 controls. There were no significant differences in gender, age, energy intake, and nutrient consumption between groups. Controls had higher BMI compared to cases (Table 1). Cases had higher percentage of Taste loss (43.3% vs 10% p = 0.04), and taste distortion (33.3 vs 10%, p = 0.05) (Table 2).

**Table 1:** Descriptive statistics in cancer patients in the second chemotherapy cycle and controls

Variable	Cases (*n *= 30)	Controls (*n *= 30)	*p*
Gender			
Male	53%	40%	0.3
Female	47%	60%	
Age (years) (mean ± SD)	56.0 ± 15	49.4 ± 11	0.07
BMI	21.6 ± 1.4	25.1 ± 0.9	**0.02**
Calories/day	1,830 ± 732	1,621 ± 717	0.26
Proteins (g/day)	69 ± 42	57.4 ± 26	0.21
Carbohydrates (g/day)	221 ± 88	202.5 ± 105	0.46
Fat (g/day)	75.9 ± 33	63 ± 36	0.15
Zinc (mg/day)	15.3 ± 7.9	16.7 ± 10	0.56
Alcohol (g/day)	1.06 ± 0.6	1.18 ± 0.9	0.82
Caffeine (g/day)	59 ± 66	146.8 ± 230	0.61*
Smoking	3%	16.6%	0.19

BMI = body mass index

*Mann-Whitney *U *test. SD, Standard deviation

**Table 2:** Subjective chemosensory complaints in cancer patients in the second chemotherapy cycle and controls.

Subjective symptoms	Cases	Controls	p *
Taste loss	13 (43.3%)	3 (10%)	**0.04**
Taste distortion	10 (33.3%)	3 (10%)	**0.05**
Bad taste in the mouth	17 (56.6%)	7 (23.3%)	0.06

* Chi squared test

We found significant differences in DT of sweet flavor (*p *= 0.05) and bitter RT (*p *= 0.04) between cases and controls (Table 3). When the control and test group were matched, the sweet DT presented statistical significance (p = 0.027). Patients under chemotherapy required increased concentrations of taste flavors in order to recognize bitter- and sweet- flavor. We correlated the upper and lower values from the median of the two significant threshold values (sweet DT and bitter RT, since umami did not show significant differences between groups) with the median of energy and nutrients intake. Cases with higher than the median sweet DT presented significant lower calorie consumption per day (1,450 vs. 1,970 kcal/day), as well as lower nutrient consumption: protein (53 vs. 74 g/day), carbohydrate (167 vs. 240 g/day), and zinc (9.6 vs. 17 mg/day) (Table 4). Patients with values higher than the median for bitter RT had significantly lower calorie (1,493 vs. 2,124 kcal), protein (52 vs. 83 g/day), carbohydrate (182 vs. 254 g/day), and fat (62 vs. 87 g/day) consumption (Table 5). No differences were found in detection or recognition thresholds and nutrient intake between the control group.

**Table 3:** Median of Detection and recognition thresholds in cancer patients in the second chemotherapy cycle and controls

Threshold	Cases (μmol/ml)	Controls (μmol/ml)	p *	*Mean rank differences (z)*	p**
Sweet DT	6.4 ± 4	4.4 ± 1.7	**0.05**	**2.22**	**0.027**
Sweet RT	8.9 ± 4	8.1 ± 5.8	0.58	0.98	0.325
Bitter DT	92 ± 5.7	89.3 ± 16	0.30	0.85	0.398
Bitter RT	100 ± 8	95 ± 7	**0.04**	**0.63**	0.527
Umami DT	1.1 ± 0.8	0.84 ± 0.6	0.19	0.04	0.96
Umami RT	2.1 ± 0.7	1.9 ± 0.7	0.3	0.10	0.919

μmol/ml = micromol/millimeter

*Mann-Whitney *U *test

** Wilcoxon Signed Ranks test

**Table 4:** Median of sweet detection threshold vs. diet consumption in cancer patients in the second chemotherapy cycle

Nutrient	≥ 6.4 μmol/ml	<6.4 μmol/ml	p*
Calories per Day	1,450 ± 833	1,970 ± 658	**0.05**
Proteins (g/day)	53 ± 32	74 ± 45	**0.02**
Carbohydrates (g/day)	167 ± 81	240 ± 84	**0.04**
Fat (g/day)	57 ± 33	82 ± 31	0.08
Zinc (mg/day)	9.6 ± 5.4	17 ± 7	**0.02**

g = grams

mg = milligrams

μmol/ml = micromole/millimeter

* Mann-Whitney *U *test

**Table 5:** Median of Bitter recognition threshold vs. diet consumption in cancer patients in the second chemotherapy cycle

Nutrient	≥ **100 μmol/ml**	<100 μmol/ml	p*
Calories per Day	1,493 ± 452	2,124 ± 812	**0.01**
Proteins (g/day)	52 ± 17	83 ± 53	**0.04**
Carbohydrates (g/day)	182 ± 58	254 ± 98	**0.02**
Fat (g/day)	62 ± 22	87 ± 38	**0.03**
Zinc (mg/day)	13.2 ± 6	17 ± 9	0.18

g = grams

mg = milligrams

μmol/ml = micromole/millimeter

* Mann-Whitney *U *test

The proportion of patients that were not able to complete their daily energy requirements was higher in those with values of sweet DT, sweet RT, and bitter RT above the median compared to patients with normal taste thresholds (Table 6). Lower daily caloric intake was found in patients with values above the median of sweet DT (-632 ± 361 vs -32 ± 162 kcal/day, p = 0.05), sweet RT (-428 ± 2.58 vs 14.7 ± 8.2 kcal/day, p = 0.06) and bitter RT (-487.4 vs 66 kcal/day, p = 0.031). The percentage of subjects with weight loss was higher in patients with bitter RT above the median (100% vs 67%, p = 0.03). Umami taste thresholds differences were not associated with changes in energy and nutrient consumption.

**Table 6:** Proportion of cases that were not able to complete their daily energy requirements was higher between patients with sweet and bitter DT and RT above the median compared to patients with normal taste thresholds

Threshold	Energy requirement (%)		p	Energy requirement	p
	No	Yes		Media ± SD	
**Sweet DT**					
≥ 6.4 μmol/ml	87.5	12.5	0.03*	-632 ± 361	**0.05**
<6.4 μmol/ml	40.9	59.1		-32 ± 162	
Sweet RT					
≥ 8.9 μmol/ml	83.3	16.7	**0.05**	-428 ± 2.58	0.06
<8.9 μmol/ml	37.5	62.5		14.7 ± 8.2	
Bitter DT					
≥ 92 μmol/ml	66.7	33.3	0.65*	-487.4 ± 211	**0.03**
<92 μmol/ml	50	50		66.0 ± 215	
Bitter RT					
≥ 100 μmol/ml	66.7	33.3	**0.05**	-208 ± 188	0.82
<100 μmol/ml	50	50		-126 ± 162	

**Threshold**	**Protein requirement (%)**		**p**	**Protein requirement**	**p**
	**No**	**Yes**		**Media ± SD**	

**Sweet DT**					
≥ 6.4 μmol/ml	55.6	44.4	0.76	-15.6 ± 13.5	0.25
<6.4 μmol/ml	50	50		7.9 ± 9.5	
Sweet RT					
≥ 8.9 μmol/ml	71.4	28.6	0.06	-12.2 ± 6.6	0.07
<8.9 μmol/ml	37.5	62.5		13.7 ± 12.4	
Bitter DT					
≥ 92 μmol/ml	66.7	33.3	1.0*	-13.2 ± 7.6	0.19
<92 μmol/ml	50	50		14.6 ± 12.7	
Bitter RT					
≥ 100 μmol/ml	43.8	56.3	0.26	1.4 ± 9.7	0.9
<100 μmol/ml	64.3	35.7		2.5 ± 11.3	

DT = detection threshold

RT = recognition threshold

SD = standard deviation

μmol/ml = micromole/millimeter

Mann-Whitney *U *test

* Fisher' s Exact Test

## Discussion

Abnormalities in taste recognition have been reported in patients with cancer [29-34]. In our study, cancer patients reported more subjective chemosensory complaints than controls. In addition, these differences could not be attributed to age or gender differences because demographic characteristics and performance status were similar in both groups. The most frequent complaint was taste loss and a tendency toward bad taste perception. Similar results were found in other studies: in a study made by Hutton et al. [35], 86% of patients with cancer reported some degree of subjective chemosensory abnormalities. In another study that included 284 cancer patients, the most frequently chemosensory complaints were dry mouth, decreased appetite, nausea, and vomiting [18].

Berberetche et al. [36] reported 110 cancer patients with significantly higher taste thresholds, in the four basic flavors than controls, detected by electrotaste test. Our study found significant differences in bitter RT and sweet DT between cancer patients under chemotherapy treatment and controls. Other studies have found similar results, Carson et al. [29] reported slight increases in sour DT and bitter RT during the early chemotherapy treatment period and normalization of the sweet RT after 2 weeks of treatment. In a study that included 30 Lung cancer patients and controls, differences in recognition thresholds for sweet taste were assessed [30]. Trant et al. [37] reported that 22 patients under chemotherapy were less likely to display a distinct preference for any of the five concentrations of sucrose, particularly high levels, than those not on chemotherapy.

In this study, no differences were found in umami taste RT between groups. Subjects were not able to describe the flavor; this is why some subjects had no detection or recognition threshold at any concentration. There is only one study in the literature that determined umami DT in cancer patients, this study included 30 patients with head and neck cancer under radiotherapy; significantly impaired DT of umami taste was revealed at 30 Gy detected by electrotaste test [24]. No studies of umami taste detection or recognition thresholds conducted in patients under chemotherapy were found in the literature.

During ingestion and digestion processes, sensory information is transmitted to the brain and integrated with past food memory and the hunger/satiety conditions. If the meal is experienced an unsatisfactory event, such as abdominal pain after eating, it is recorded in the memory as unpleasant (aversive) food [27]. Taste disorders are unpleasant experiences; it is well known that taste signals affect food preference and food intake, playing an important role in anorexia, weight loss and malnutrition disorders in patients with cancer, however, there are few studies determining the impact of taste abnormalities and food intake in patients with cancer.

Calories and nutrient intake did not show statistical differences between cases and controls in our studied population, this could be due to a good performance status in all included patients (ECOG 0-1) and the exclusion of individuals with gastrointestinal cancer.

The comparison between patients with upper sweet DT and bitter RT values above the median versus those with values below the median, showed a significant difference for low calorie and nutriment intake in cancer patients (cases) with higher sweet DT and bitter RT values. (Tables 4, and 5). These findings agree with other reports and confirm that taste disorders not only reduce the patient's quality of life (QOL), but they may also lead to insufficient eating habits affecting dietary intake and nutritional status [15,38,39]. Hutton et al [35] reported lower energy intake (by 900-1,000 kcal/day), higher rates of weight loss, and lower QOL scores in patients with severe chemotherapy-associated chemosensory distortions. Other study including 72 patients under chemotherapy, reported changes in consumption of sweet and salty foods with 82% of the patients avoiding food with such flavors [40].

Zinc deficiency has been associated with taste disorders in some but not all reported studies [41-46]. A possible explanation has been described: Drugs that cause hypogeusia have a sulfhydryl group in their structures; this component is known to bind and chelate heavy metal ions like zinc [16]. In the present study, zinc consumption was not significantly different between cases and controls. However, cases with higher sweet RT exhibited significantly less zinc consumption than patients with lower thresholds; these data might suggest some relationship between high sweet RT and low zinc intake.

More measures are needed to prevent taste distortions in patients with cancer. Clinicians play an important role in the detection, education, and referring of patients who experience taste disorders. Nutritional management of individuals with chemosensory disorders requires a complete clinical and nutritional evaluation with appropriate dietary-intake measurements and expert nutritional counseling.

Methodological weaknesses of this research include: Variability in cancer types and chemotherapy drugs used; and the absence of basal taste disorder evaluation before chemotherapy treatment, and for establishing a causal association between chemotherapy and taste disorders. Continuing research is required to develop better understanding of the nature, frequency, severity, and duration of taste alterations and their significance in food consumption and malnutrition in those patients with cancer under chemotherapy.

## Conclusions

Cancer patients under chemotherapy have higher sweet detection threshold and higher bitter recognition threshold compared to patients without cancer. These abnormalities are associated with decreased calorie, protein, carbohydrate, fat and zinc intake; there were also a high proportion of patients that were not able to complete their daily energy requirements, resulting in weight loss. Taste disorders could signify an important factor in malnutrition and wasting of patients with cancer.

## Competing interests

The authors declare that they have no competing interests.

## Authors' contributions

KS and CR contributed to study data collection. KS, OA, AL, DG, DM contributed to sample process and manuscript writing. KS, RS and OA contributed to this study design and analysis and manuscript writing. All authors read and approved the final manuscript.
